# Improving access to and use of maternal health services during COVID-19: Experience from a health system strengthening project in Guinea

**DOI:** 10.3389/fpubh.2022.1004134

**Published:** 2022-10-13

**Authors:** Mariama Kouyate, Lansana Barry, Abdoulaye Sow, John De Maesschalck, Willem Van De Put, Sidikiba Sidibé, Norohaingo Adrianaivo, Delphin Kolié, Alexandre Delamou

**Affiliations:** ^1^African Center of Excellence for the Prevention and Control of Communicable Diseases, Gamal Abdel Nasser University of Conakry, Conakry, Guinea; ^2^National Center for Training and Research in Rural Health of Maferinyah, Forecariah, Guinea; ^3^Institue of Tropical Medicine, Antwerp, Belgium; ^4^Fraternité Médical Guinée, Conakry, Guinea; ^5^Memisa, Bruxelles, Belgium

**Keywords:** COVID-19, maternal health, antenatal care, institutional delivery, Guinea

## Abstract

The purpose of this study was to document the experience of health providers' capacity strengthening during health crises and the contribution of such to the health system and the population resilience in the face of the COVID-19 pandemic in Guinea. We conducted a cross-sectional study using routine data collected from 41 health facilities in the project intervention areas, including associative health centers, community health centers, and district hospitals,. These data covered the period between 2019 and 2021. Results showed that all the community health centers (CMCs) had a clean internal and external environment, compared to health centers (95.2%) and district hospitals (33.3%). Hand washing was systematic among visitors attending CMCs and district hospitals (HPs). However, 28.6% of visitors attending associative health centers (AHCs) did not wash their hands. Temperature taking for visitors was not carried out in all CMCs and in 90.5% of the AHCs; unlike in the HC and HP where the temperature of each patient was taken before entering the consultation room. The obligation to wear masks was higher in the HP and in the HC, compared to the CMC and AHC where the order of non-compliance with the wearing of masks was, respectively 36.4 and 19%. Non-compliance with social distancing in the waiting rooms and between users was observed in all facilities. The project's interventions mainly contributed to improving the utilization of prenatal consultation and institutional delivery services; the beginning of the interventions was marked by an increase of an average of 17 ANC1 per month in CMCs and 116 ANC1 in health centers. Ongoing training on capacity strengthening for providers in infection prevention and control, followed by the offering of delivery kits and materials during epidemics, would contribute to the improvement and utilization of health facilities by the population.

## Introduction

The new coronavirus disease (COVID-19) emerged in China in mid-December 2019 and then spread rapidly worldwide, resulting in more than 552 million confirmed cases and 6 million deaths as of July 2022. According to recent World Health Organization (WHO) estimates, major disruptions in the utilization of maternal health services have been observed in 40% of countries in sub-Saharan Africa (SSA) (1). Authors have reported a decline in maternal health indicators between 3–12%,—including antenatal care, and institutional deliveries—during COVID-19 in eight SSA countries (2). Another study in Rwanda found that health facilities in rural areas were the most affected by the decline in the utilization of antenatal care services and institutional deliveries (3).

In Guinea, the first case of COVID-19 was recorded on March 12, 2020. As of 21 July 2022, a total of 724,638 confirmed cases and 783 deaths have been recorded nationwide (4). The country was the epicenter of the West African Ebola epidemic in 2014/2016. This epidemic had a drastic effect on maternal health services, particularly in rural areas (5–7). The main reasons for this decline in utilization of health services included people's fear of contracting the disease in health facilities; and the closure of health facilities due to the death of health workers or lack of personal protective equipment (8). Like the Ebola epidemic, the COVID-19 pandemic could lead to disruptions in the use of maternal services, particularly for populations living in rural areas (9).

However, an analysis of health services utilization during the first month of the COVID-19 pandemic declaration in Guinea (April 2020), showed a sharp decrease in first and subsequent outpatient visits in Conakry as well in the regions of Kindia, Mamou, and Labé where cases were reported (source SNIS).

To reduce the effects of the COVID-19 pandemic on the use of maternal health services, a project entitled “Strengthening the Health System to Ensure Continuity of Services and Access to Care for Vulnerable Populations in the Context of COVID-19” was implemented in Guinea. This 23-month project was piloted by the NGOs Memisa (Belgium) and Fraternité Médicale Guinée (FMG) in 41 health facilities across 4 administrative regions of the country (Conakry, Kindia, Mamou, and Labé).

In Guinea, Conakry was and continued to be the epicenter of the COVID-19 pandemic. The Health system in the capital is characterized by a proliferation of informal health facilities. This proliferation is sustained by the mismatch between health providers' supply and employment capacities of the government, as well as poor health system governance. In addition, the health system in Conakry is also characterized by a lack of communication that hinders the complementarity of actors in their common goal (improving the quality and accessibility of health care and services).

In the project areas, four (4) main interventions were undertaken:

- Training of health personnel in infection prevention and control, primary health care in emergencies including reorganization of care and patient flow;
- Providing health facilities with infection prevention and control equipment (masks, hydroalcoholic solutions, hand washing kits, personal protective equipment, etc.) and delivery equipment (delivery tables, carts, delivery boxes, etc.,).
- The provision and installation of incinerators and boreholes in health facilities;
- The provision of delivery kits (buckets, soap, clothes for newborns, etc.,) to women giving birth in health facilities.

This study was therefore undertaken to document the project's contribution to strengthening the resilience of the health system and the population during the COVID-19 pandemic. Specifically, this study aimed to:

- Describe the practices of health care providers with respect to ICP after the implementation of the Memisa health system strengthening project interventions.
- Analyze the effects of the Memisa project interventions on maternal health indicators (ANC1, ANC4 and institutional delivery), describing and comparing the period before COVID-19, during COVID-19 and the intervention.

The results of such a study could guide future public health interventions to improve the utilization of health services by populations in health emergency context.

## Materials and methods

### Study design

This was a cross-sectional study using routine data from maternal health services, and blinded observations of health care workers regarding the implementation of infection prevention and control (IPC) measures. Routine data covered the pre-COVID-19 period (March 2019 to February 2020), COVID-19 and pre-intervention period (March 2020 to March 2021), and COVID-19 and intra-intervention period (April 2021 to December 2021).

### Study setting

#### General setting

Guinea is located in West Africa, with a population of over 12 million people (10) and a literacy rate of 31% for women and 55% for men. Women make up 53 % of the general population and those of childbearing age make up 45 % of the total female population. The country has high maternal and neonatal mortality rates with 576 maternal deaths per 100,000 live births and 31 neonatal deaths per 10,000 live births in 2017 (11). The total fertility rate is estimated at 4.8 children per woman with a total fertility rate of 165 births per 1,000 women of childbearing age per year (10).

Guinea has 8 health regions (Conakry, Kindia, Labe, Mamou, Boke, Kankan, Faranah, and N'zérékoré) divided into 38 health districts, 33 of which are rural. The country's health pyramid is structured into three distinct levels of care: primary, secondary, and tertiary. The primary level includes 414 government health centers, and a dozen community medical cabinets and associative health centers; the secondary level includes 4 communal medical centers, 26 district hospitals, and 7 regional hospitals; and the tertiary level includes 3 national or reference hospitals.

### Maternal health service delivery in Guinea

Maternal health services in Guinea's health facilities are aligned with international guidelines for quality care (12). These guidelines define minimum packages of maternal health services by type of health facility. For example, primary health care facilities provide antenatal care (ANC) and eutocic deliveries. Emergency obstetric care for complicated deliveries (including cesarean sections) is required for secondary and tertiary health facilities. In addition, at least four ANC visits are recommended for each pregnant woman and at least 90% of all deliveries should be performed in health facilities (13). In addition, national guidelines recommend that qualified health personnel, including midwives, conduct deliveries in health facilities doctors, nurses, and technical health workers.

### Specific setting

The health facilities in the intervention zones of the “Strengthening the health system to ensure continuity of services and access to care for vulnerable populations in the context of COVID-19 in Guinea” project served as the setting for this study. 41 health facilities, including 32 in the private sector and nine in the public sector, in four health regions, benefited from the interventions of the above-mentioned project. These health facilities are distributed as follows: 11 community medical cabinets; 21 associative health centers in the city of Conakry; six public health centers, two district hospitals (Pita and Télimélé) and the regional hospital of Labe.

### Population and period of study

Quantitative data collection focused on women who used maternal health services between March 2019 and December 2021 in all facilities in the intervention zones and facility observations on infection prevention and control measures. Data were collected over a three (3) week period from January 23 to February 11, 2022.

### Sampling

**Figure 1:**
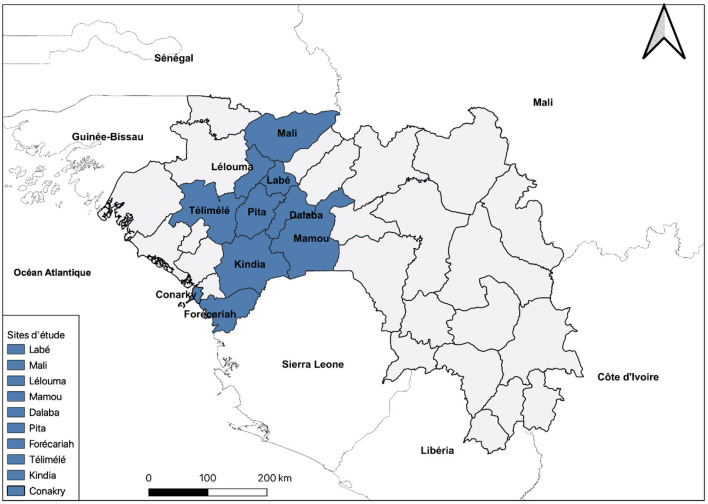
Health districts in the region of Kindia, Mamou, Labé and Conakry, Guinea, included in the study.

The sampling was exhaustive; all the health facilities in the intervention zone and benefiting from the project intervention were selected for data collection. These were 41 health facilities, including 11 COMEC-Gui community medical cabinets; 17 associative health centers in the city of Conakry of the Actions Concertées pour la Santé (ACS) network; Maferinyah health center, the HCs of Pita and Télimélé, Labe regional hospital, and the district hospitals (Pita and Télimélé) (Figure 1).

### Data collection

Routine data on maternal health indicators were extracted from the district health information system (DHIS2) for each of the health facilities concerned. However, to ensure good data quality and reduce bias due to missing data, the monthly reports of the health facilities concerned were also used. Data extraction from the two sources mentioned above was done using forms previously established for this purpose. Both data sources were used to minimize the missing data sometimes encountered in the DHIS2. We did not compare the data from the two sources.

An observation of the providers' practices and the internal and external environment of the facilities was carried out using an observation grid. This observation grid was composed of 18 measures of infection prevention and control. These measures could be categorized into two main themes: patients' safety and security; and facility hygiene. Patient safety and security categories comprised (Is there an area in the facility for sorting incoming patients, are the providers wearing the correct PPE such as gowns, masks, gloves). Meanwhile, facility hygiene was composed of (Is the external and internal environment of the facility clean, Is there running water in the facility) applications, including the assessment of the internal and external environment, patient sorting areas, the presence of handwashing devices at the entrance of the facilities, the use of handwashing kits by visitors to the health facilities, the taking of temperatures by visitors upon entering the facility, the wearing of masks by patients and health care providers, physical distancing, and waste sorting. These observations took place approximately 12 months after the providers were trained in IPC. The observation grids were administered by a multidisciplinary team (composed of two doctors and a sociologist) previously trained in data collection tools. Data collection took place over a period of three (3) weeks, from January 23 to February 11, 2022.

### Data management and analysis

We processed and tabulated the data using Microsoft Office Suite Excel, and then analyzed using Stata version 16 software (Stata Corporation, College Station, TX, United States). Data for each indicator were clustered into a panel for exploitation.

outine data from March 2019 to December 2021 from the 41 facilities were collected over the same period and these quantitative data were described using proportions with their 95% confidence intervals. Trends in indicators before COVID-19 and during COVID-19 and during intervention were estimated by component using interrupted series analysis, as appropriate. We used segmented regression to measure changes in level and trend that followed the occurrence of COVID-19 and the intervention. Conveniently, we referred to Linden et al. (14) paper, which presents the itsa command and the effect of an intervention on an outcome variable for a given period.The Itsa (Interrupted time-series analysis for single and multiple groups with multiple panels) command on Stata was used to estimate the causal effect of the pandemic on a potential decline in health service use. The Itsa method therefore compares the finding that would have been by extrapolating the trend line of the finding of the period before the pandemic, as if it had never happened. Itsa uses ordinary least squares (OLS) and its use assumes that the observation point data are reported as panel data.

A modeling approach was used to assess how the average number of users of each healthcare facility changed immediately after the first COVID-19 cases were recorded, i.e., in March 2020, but also from the start of the project interventions, i.e., in April 2021 (change in level) and gradually over time (change in slope). The data were grouped by type of facility: private (associative health centers and community medical cabinets) and governmental (health centers and hospitals) in the four regions of the intervention areas. To facilitate the analysis, this regression model was used for each indicator: *Yt* = β_0_+β_1_*T*_*t*_+β_2_*X*_*t*_+β_3_*X*_*t*_*T*_*t*_+∈_*t*_, where β0 represents the intercept or intercept or initial level, β1 is the change in the variable of interest (Yt) for 1 unit time, β2 represents the immediate change in Yt following the intervention, β3 represents the change in the trend of Yt before COVID-19 relative to the trend before intervention (change over time) εt the error term:

The data from this matrix were grouped according to their similarity and difference and then described to assess the level of compliance with infection prevention and control measures in the health facilities.

### Ethical considerations

The research protocol for this study was approved by the National Health Research Ethics Committee of Guinea (number L-080-CNERS-21) before the start of data collection. Then, an authorization had been obtained at the level of the health facilities before the beginning of the data collection including aspect of confidentiality.

## Findings

### Infection prevention and control practices of health care providers

The observation of the internal and external work environment showed that 100% of the community medical cabinets (CMCs), 95.2% of the associative health centers (AHCs) and health centers (HCs) were clean. In contrast, only 33.3% of hospitals (HP) had a clean internal and external environment. Analysis of the observation data revealed the existence of sorting areas in 83.3% of the HCs, 72.7% of the CMCs and 66.7% of the hospitals, compared with 57.1% of the AHCs.

It was found that hand washing devices at the entrance of the facilities were functional in 83.3% of the HCs, 72.7% of the CMCs, 66.7% of the AHCs and 33.3% of the hospitals. According to the results of the observations made, hand washing of visitors before admission to the facilities was systematic in the CMCs and HPs. However, 28.6% of the visitors in AHCs did not wash their hands before admission to the facility.

Temperature taking for visitors was not done in all the CMCs visited (100%) and in 90.5% of the AHCs; unlike in the HC and HP where the temperature of each patient was taken before entering the consultation room.

In addition, all health care providers observed in hospitals and HCs respected the mandatory wearing of masks compared to those in CMCs (36.4%) and AHCs (19%). The observation revealed that physical distancing in the waiting rooms and between users was not respected in all the facilities visited.

There was a waste sorting mechanism in all the hospitals visited, compared to 83.3%, 81% and 72% of the HC, AHC and CMC, respectively. It should be noted that in the prefectural hospital of Pita, empty cartons were used instead of safety boxes, which had been out of order for several months. Open burning of waste occurred in 52.4% of the AHCs. Lack of running water was observed in 54.5% of CMCs and 14.3% of AHCs (Table 1).

**Table 1:** Analysis of the observation matrix for the application of the IPC's on health structures. June 2022.

**Application of IPC measures**	**Associative health centers**		**Community medical**		**Government health centers**		**Hospitals**	
			**Cabinets**			
	***N***	**%**	***N***	**%**	***N***	**%**	**N**	**%**
**Hygiene of health facilities**								
Is the external and internal environment of the facility clean?								
Yes	20	95.2	11	100	20	95.2	1	33.3
No	1	4.8	0	0	1	4.8	2	66.7
**Is there a functional hand washing device at the entrance to the facility?**								
Yes	14	66.7	8	72.7	5	83.3	1	33.3
No	7	33.3	3	27.3	1	16.7	2	66.7
**Do providers respect hand washing between procedures?**								
Yes	12	57.1	7	63.6	6	100	1	33.3
No	9	42.9	4	36.4	0	0	2	66.7
**Do all visitors wash their hands before entering the center?**								
Yes	6	28.6	8	72.7	6	100	3	100
No	15	71.4	3	27.3	0	0	0	0
**Is there a tap water supply in the structure?**								
Yes	18	85.7	5	45.5	6	100	3	100
No	3	14.3	6	54.5	0	0	0	0
**Do providers wash their hands properly according to the guidelines?**								
Yes	6	28.6	8	72.7	6	100	3	100
No	15	71.4	3	27.3	0	0	0	0
**Wearing a ring on the fingers**								
Yes	5	23.8	0	0	0	0	3	100
No	16	76.2	11	100	6	100	0	0
**Presence of fingernails**								
Yes	2	9.5	0	0	0	0	3	100
No	19	90.5	11	100	11	100	0	0
**Is the waste area clean and tidy?**								
Yes	16	76.2	9	81.8	6	100	3	100
No	5	23.8	2	18.2	0	0	0	0
**Patient safety**	
**Is the temperature taken for all visitors to the center?**								
Yes	2	9.5	0	0	6	100	3	100
No	19	90.5	11	100	0	0	0	0
**Do the providers wear PPE correctly (Blouses, masks, gloves)?**								
Yes	7	33.3	9	81.8	6	100	2	66.7
No	14	66.7	2	18.2	0	0	1	33.3
**Do providers and users respect the mandatory wearing of masks?**								
Yes	4	19.0	7	63.6	6	100	3	100
No	17	81.0	4	36.4	0	0	0	0
**Is physical distance respected in the waiting rooms between users?**								
Yes	5	23.8	4	36.4	0	0	0	0
No	16	76.2	7	63.6	6	100	3	100
**Is physical distance maintained in the consultation and care offices between providers?**								
Yes	16	76.2	8	72.7	6	100	2	66.7
No	5	23.8	3	27.3	0	0	1	33.3
**The waste is buried or incinerated and not burned in the open air?**								
Yes	10	47.6	10	90.9	6	100	3	100
No	11	52.4	1	9.1	0	0	0	0
**There are trash cans and safety boxes in all areas where waste is produced?**								
Yes	17	81.0	8	72.7	5	83.3	3	100
No	4	19.0	3	27.3	1	16.7	0	0
**The trash cans are well labeled and the waste segregation is respected?**								
Yes	9	42.9	9	81.8	4	66.7	3	100
No	12	57.1	2	18.2	2	18.2	0	0
**Is there a triage area in the facility for incoming patients?**								
Yes	12	57.1	8	72.7	5	83.3	2	66.7
No	9	42.9	3	27.3	1	16.7	1	33.3

### Use of maternal health services before COVID-19, before and after intervention

The interrupted series analysis approach used allowed us to highlight the trend in the use of maternal health services in the pre-COVID-19 period, before and during interventions in the intra-COVID-19 period. In all, the data collected covered 34 months: 12 months for the pre-COVID-19 period (March 2019-February 2020); 13 months for the intra-COVID-19 period (March 2020-March 2021) and before the interventions; and 9 months for the intra-COVID-19 period and during the intervention (April 2021-December 2021).

### First antenatal care visit (ANC1)

**Figure 2:**
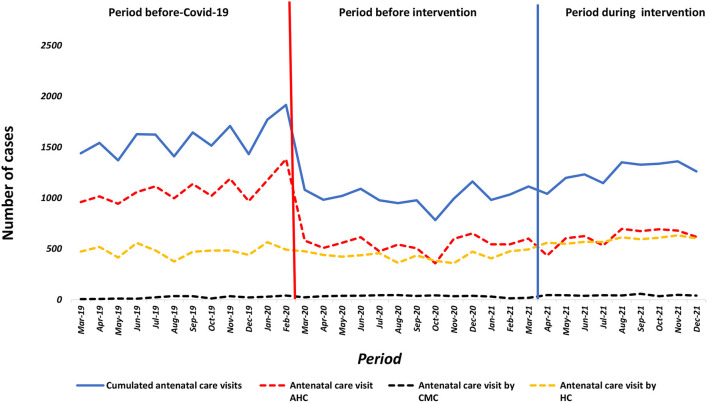
Number of women receiving one antenatal AHC, CMC, HC Guinea from March, 2019 to December, 2021.

As soon as the first COVID-19 cases were reported in March 2020, a drastic decline in ANC1 service utilization was observed in the associative health centers [β = −702; 95% CI = (−885; −520); *p* = 0.001] and the HCs [β = −64; 95% CI = (-137; 9); *p* = 0.082], while no changes were observed in the CMC. At the beginning of the project interventions in April 2021, this decrease continued in the AHCs, in contrast to the CMCs which experienced a significant increase of an average of 17 ANC1 per month [β = 17; 95% CI = (-3; 31); *p* = 0.021] similarly to the HCs where we saw an increase in the monthly average with 116 ANC1 [β = 116; 95% CI = (52; 180); *p* = 0.001] (Figure 2).

A Comparison of the pre-COVID-19 period to the intervention period showed a statistically significant increase in the average monthly number of ANC1 in AHCs and HCs in contrast to CMCs where it was zero (Table 2).

**Table 2:** Estimates of parameters for monthly utilization of first antenatal visits. AHC, CMC, HC, Guinea. June 2022.

**First antenatal visits**									
**Private facilities**							**Governmental facilities**		
**Variables**	**AHC**			**CMC**			**HC**		
	**Coef**	**95%**	***P*** **value**	**Coef**	**95%**	***P*** **value**	**Coef**	**95%**	***P*** **value**
Health service coverage at the beginning of the pre-COVID-19 period (β0)	950	877; 1,024	0, 000	8	2; 14	0, 010	470	411; 530	0, 000
Average monthly change in service coverage during the pre-COVID-19 period (β1)	24	4; 43	0, 018	3	2; 4	0, 000	2	−7; 10	0, 667
Immediate change in service coverage level at the start of COVID-19 (β2)	−702	−885;−520	0, 000	0	−15; 15	0, 988	−64	−137; 9	0, 082
Difference between the trend in service coverage during COVID-19 and the pre-COVID-19 period (β3)	−21	−42;−1	0, 044	−4	−6;−2	0, 001	−1	−12; 10	0, 861
Immediate change in service coverage level at the start of the INTERVENTION (β2)	−25	−156; 105	0, 694	17	3; 31	0, 021	116	52; 180	0, 001
Difference between the trend in service coverage during the INTERVENTION and the period prior to COVID-19 (β3)	18	−4; 41	0, 104	1	−1; 3	0, 318	8	0; 16	0, 054
**Total**	
Linear trend before COVID-19 and during INTERVENTION	21	0; 41	0, 054	0	−1; 1	0, 909	9	5; 13	0, 000

### Fourth antenatal care visit (ANC4)

**Figure 3:**
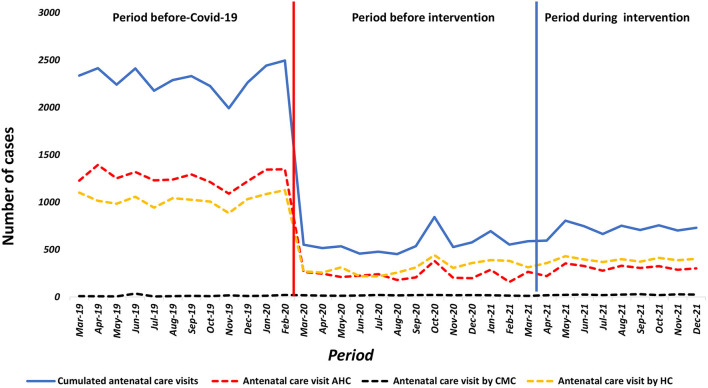
Number of women receiving four antenatal AHC, CMC, HC Guinea from March, 2019 to December, 2021.

Upon reporting of the first COVID-19 cases in March 2020, a drastic decrease in ANC4 service utilization was observed in AHCs [β = −1,015; 95% CI = (-1,146;−883); *p* = 0; 001] and HCs [β = −794; 95% CI = (-909; 678); *p* = 0.001], while it remained virtually unchanged in the CMC. At the start of the project interventions in April 2021, an increase in the average monthly number of 60 (ANC4) was observed in both AHCs and CMC, in contrast to HCs where ANC4 utilization continued to decline. However, this increase was not statistically significant (Figure 3).

A Comparison of the pre-COVID-19 period to the intervention period showed a non-significant increase in the average monthly number of ANCs4 in AHCs and HSs in contrast to CMCs where the decrease persisted (Table 3).

**Table 3:** Estimates of parameters for the monthly use of fourth antenatal visits. AHC, CMC, HC, Guinea. June 2022.

**Fourth antenatal visits**									
**Private facilities**							**Governmental facilities**		
**Variables**	**AHC**			**CMC**			**HC**		
	**Coef**	**95%**	***P*** **value**	**Coef**	**95%**	***P*** **value**	**Coef**	**95%**	***P*** **value**
Health service coverage at the beginning of the pre-COVID-19 period (β0)	1,274	1,186; 1,361	0, 000	10	−1; 20	0, 074	1,013	941; 1,086	0, 000
Average monthly change in service coverage during the pre-COVID-19 period (β1)	−2	−18; 14	0, 819	0	−1; 2	0, 397	2	−11; 15	0, 749
Immediate change in service coverage level at the start of COVID-19 (β2)	−1,015	−1,146;−883	0, 000	1	−5; 8	0, 685	−794	−909;−678	0, 000
Difference between the trend in service coverage during COVID-19 and the preCOVID-19 period (β3)	1	−16; 19	0, 871	−1	−2; 1	0, 396	9	−6; 24	0, 236
Immediate change in service coverage level at the start of COVID-19 (β2)	60	−46; 166	0, 256	4	−1; 10	0, 131	−1	−73; 71	0, 985
Difference between the trend in service coverage during the INTERVENTION and the pre-COVID-19 period (β3)	3	−12; 18	0, 688	1	0; 2	0, 142	−9	−19; 0	0, 057
Linear trend before COVID-19 and during INTERVENTION (b1+ b3)	3	−11; 16	0, 696	1	0; 1	0, 095	11	4; 18	0, 003

### Institutional deliveries

**Figure 4:**
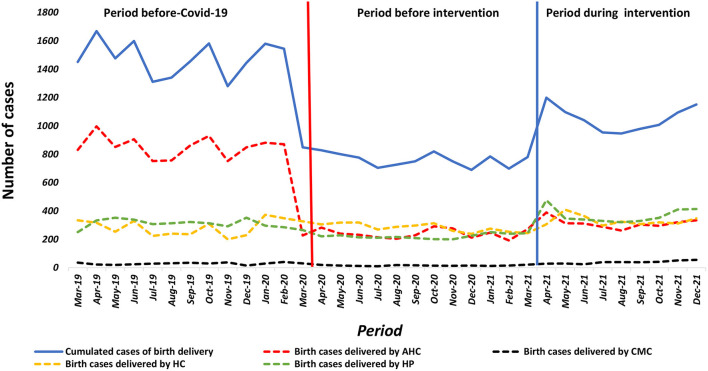
Number of women giving birth in the health facility AHC, CMC, HC Guinea from March, 2019 to December, 2021.

As soon as the first COVID-19 cases were reported in March 2020, a drastic and significant decrease in the number of institutional deliveries was observed in AHCs [β = −596; 95% CI = (-677;−516); *p* = 0.001]. It also relatively decreased in CMCs [β = −13; 95% CI = (-28; 1); *p* = 0.066] and HPs [β = −4; 95% CI = (-135;−36); *p* = 0.001] in contrast to HCs where it relatively increased (Figure 4). At the beginning of the interventions in April 2021 in the AHCs experienced a significant increase of 87 institutional deliveries on average [β = 87; 95% CI = (15; 160); *p* = 0.020], as did the CMCs where on average an increase of 11 deliveries was noted [β = 11; 95% CI = (2; 20); *p* = 0.014], as well as 105 at the HCs [β = 105; 95% CI = (40; 171); *p* = 0.003], unlike the hospitals where no significant change was seen (Table 4).

**Table 4:** Estimates of parameters for monthly utilization in institutional delivery services. AHC, CMC, HC, HP Guinea. June 2022.

**Institutional deliveries**												
**Private facilities**							**Governmental Facilities**					
**Variables**	**AHC**			**CMC**			**HC**			**HP**		
	**Coef**	**95%**	***P*** **value**	**Coef**	**95%**	***P*** **value**	**Coef**	**95%**	***P*** **value**	**Coef**	**95%**	***P*** **value**
Health service coverage at the beginning of the pre-COVID-19 period (β0)	868	775; 961	0, 000	26	17; 35	0, 000	281	223; 340	0, 000	13	266; 366	0, 000
Average monthly change in service coverage during the pre-COVID-19 period (β1)	−3	−15; 9	0, 657	1	−1; 2	0, 495	0	−11; 12	0, 951	0	−8; 6	0, 867
Immediate change in service coverage level at the start of COVID-19 (β2)	−596	−677;−516	0, 000	−13	−28; 1	0, 066	38	−56; 133	0, 413	−4	−135;−36	0, 001
Difference between the trend in service coverage during COVID-19 and the preCOVID-19 period (β3)	3	−10; 16	0, 685	−1	−3; 1	0, 317	−7	−18; 5	0, 236	0	−7;9	0, 833
Immediate change in service coverage level at the start of COVID-19 (β2)	87	15; 160	0, 020	11	2; 20	0, 014	105	40; 171	0, 003	3	45; 245	0, 006
Difference between the trend in service coverage during the INTERVENTION and the pre-COVID-19 period (β3)	−4	−16; 9	0, 555	4	2; 5	0, 000	3	−8; 14	0, 582	0	−20; 18	0, 926
**Total**	
Linear trend before COVID-19 and during intervention	−4	−15; 7	0, 514	3	2; 4	0, 000	−3	−14; 8	0, 532	0	−19; 18	0, 946

## Discussion

To our knowledge, this study is the first of its kind to analyze health care providers' practice of infection prevention and control (IPC) and maternal health service utilization during the COVID-19 pandemic in Guinea. Our study shows mitigated results of health care providers' practice with respect to the application of IPC measures. For example, almost all health care providers observed in hospitals and health centers complied with the mandatory wearing of facemasks and the systematic recording of temperatures of visitors attending the health facilities. In contrast, in community health cabinets (CMCs) and associative health centers (AHCs), more than six out of 10 health providers did not respect the mandatory wearing of a facemask. In addition, the systematic measurement of temperature was not observed among visitors attending community health cabinets (CMCs) and associative health centers (AHCs). A plausible explanation for these results would be the limited number of financial sources and actors involved in the supply of personal protective equipment, including facemasks in these health facilities. Indeed, the two types of health facilities concerned are all privately owned, which would limit the intervention of state actors and other organizations in the supply of personal protective equipment. Another important explanation for the results of this study could be that the project implementers did not take into account equity in the supply of health facilities. Indeed, the practical experience of implementing the “Strengthening the health system to ensure continuity of services and access to care for vulnerable populations in the COVID-19 context” project shows that the health facilities involved in the project were provisioned only for a few months. This inconsistent allocation of health commodities would certainly not have enabled the private health facilities to prevent input shortages. Ashinyo, et al. (15) in Ghana observed low compliance by health care providers with the use of personal protective equipment in health facilities reporting frequent input shortages. The low compliance of health care providers with regard to visitors' temperature recording could be explained by their low perception of the risk of disease transmission during the data collection period. In fact, the data collection period for this study (January-February 2022) did not correspond to a period of high intensity of COVID-19 transmission in Guinea (16). Huang et al. (17) in their longitudinal study of health care providers' behavior in France reported a reduction in healthcare providers' compliance with infection prevention and control measures during periods when containment was lifted and transmission was low. In view of these results, we recommend that the actors of this particular project, and the actors of the health system in general, take into account, in future projects, the equity of supply of inputs between public and private health structures, but also to favor periodic (monthly or semi-annual) allocation of inputs instead of temporal or single allocation.

Another mitigated result of this study is that more than half (52.4%) of the AHCs burned their waste in the open air compared to other types of health facilities that incinerated their waste. Similarly, more than four out of 10 community health cabinets did not have patients' separation or sorting areas. In our experience, the community health cabinets, all of which are located in the Conakry region, are the result of the transformation of human dwellings; therefore, they often do not meet the standards of health facilities in terms of space and location. For example, most of the community health cabinets visited during the data collection did not have space for incinerators for waste management, nor they did for patient sorting areas. The absence of sorting areas in all of the ASCs raises questions about how these health facilities proceed during the COVID-19 pandemic. Taking into account the recurrence of diseases with epidemic potential in Guinea, one might question the capacity of these health facilities to offer safe health care to the population (17). The other question about the safety of the care offered in the AHCs, given their location, particularly their proximity to people's homes, is the practice of burning waste in the open air in some of these facilities. Indeed, the probability of these health facilities practicing open burning of biomedical waste to release pathogenic bacteria and toxic gases into their environment would be high (18, 19). These results point to the need for health system actors to support associative health centers in obtaining appropriate space for their establishment, in order to ensure safe health care delivery during epidemics (patient sorting areas) as well as safe management of biomedical waste from these health structures.

Our analysis showed an increase in maternal health service utilization levels during the project intervention, compared to the pre-intervention period. However, these utilization levels during the project intervention period remained below pre-COVID-19 levels. These results are superimposed on the 2017 study by Delamou, et al. (5). after the Ebola outbreak in Guinea; the authors reported low levels of maternal health service utilization 12 months after the Ebola outbreak, compared with the pre-Ebola period. A key question to ask is why maternal health indicators are struggling to recover to pre-COVID-19 levels despite low lethality. These results raise questions about the resilience of the national health system, particularly the ability of health services to recover a few moments after the occurrence of epidemics (20–22). Critical analysis of health service utilization levels during the project intervention period, compared to pre-COVID-19 levels, suggests that the project's objectives were only partially achieved. One possible explanation for this finding is the delay in the provision and delivery of project inputs to beneficiary health facilities. Indeed, it was noted during data collection that some health facilities had not yet been provided with medical kits and materials. Finally, our data show that the levels of increase in maternal health services varied according to the type of health facility and the maternal health indicators covered by our study. For example, ANC4 utilization levels remained almost unchanged during the project intervention period compared to the pre-intervention period in all types of health facilities included in our study. However, levels of utilization of health services for childbirth increased statistically significantly during the intervention period, compared to the pre-intervention period, in primary health facilities (AHC, CMC, and HC). In contrast, hospitals did not experience a statistically significant increase in the level of deliveries. The increase in the level of utilization of delivery services in all primary health facilities, compared to the level of utilization of ANC4 services, could be explained by the offering of incentives such as buckets, soaps, and newborn clothes to women delivering in the health facilities. However, the small change in the level of utilization of delivery services in hospitals would be related to the low use of pregnant women in these facilities dedicated to receiving complicated cases of childbirth.

### Limitations

A limitation of this study is that due to the methodological approach used in our study, it was difficult to link changes in the levels of use of maternal health indicators during the period of the project interventions to the interventions.

## Conclusion

This study revealed a slight increase in the demand for maternal health services, which could be explained by the provision of equipment and delivery kits to support women giving birth in these health facilities. In addition, a lack of infection prevention and control measures in health centers, particularly those run by associations (AHC), has been observed. It is therefore necessary to ensure that infection prevention and control measures are respected, but also that sorting areas for patients and waste disposal circuits are set up in these different structures.

## Data availability statement

The original contributions presented in the study are included in the article/supplementary material, further inquiries can be directed to the corresponding author.

## Ethics statement

The studies involving human participants were reviewed and approved by National Ethics and Health Research Committee of Guinea. Written informed consent for participation was not required for this study in accordance with the national legislation and the institutional requirements.

## Author contributions

The study protocol was developed by MK and LB and reviewed by WD, SS, and AD. The data were analyzed by MK and DK. The first draft of the manuscript was written by MK and LB and critically reviewed by WD, JD, SS, and AD. All authors participated in the interpretation, read and approved the final version of this manuscript.

## Funding

The study was funded by the European Union.

## Conflict of interest

The authors declare that the research was conducted in the absence of any commercial or financial relationships that could be construed as a potential conflict of interest. The reviewer KK declared a shared affiliation with four of the authors to the handling editor at the time of the review.

## Publisher's note

All claims expressed in this article are solely those of the authors and do not necessarily represent those of their affiliated organizations, or those of the publisher, the editors and the reviewers. Any product that may be evaluated in this article, or claim that may be made by its manufacturer, is not guaranteed or endorsed by the publisher.
